# Individual recognition of opposite sex vocalizations in the zebra finch

**DOI:** 10.1038/s41598-017-05982-x

**Published:** 2017-07-17

**Authors:** Pietro B. D’Amelio, Milena Klumb, Mauricio N. Adreani, Manfred L. Gahr, Andries ter Maat

**Affiliations:** Max Planck Institute for Ornithology, Department Gahr - Behavioural Neurobiology, Seewiesen, 82319 Germany

## Abstract

Individual vocal recognition plays an important role in the social lives of many vocally active species. In group-living songbirds the most common vocalizations during communal interactions are low-intensity, soft, unlearned calls. Being able to tell individuals apart solely from a short call would allow a sender to choose a specific group member to address, resulting in the possibility to form complex communication networks. However, little research has yet been carried out to discover whether soft calls contain individual identity. In this study, males and females of zebra finch pairs were tested with six vocalization types - four different soft calls, the distance call and the male song - to investigate whether they are able to distinguish individuals of the opposite sex. For both sexes, we provide the first evidence of individual vocal recognition for a zebra finch soft unlearned call. Moreover, while controlling for habituation and testing for repeatability of the findings, we quantify the effects of hitherto little studied variables such as partners’ vocal exchange previous to the experiment, spectral content of playback calls and quality of the answers. We suggest that zebra finches can recognize individuals via soft vocalizations, therefore allowing complex directed communication within vocalizing flocks.

## Introduction

Within a social context, such as a cocktail party, to recognize your spouse’s voice might be of survival importance. The capacity to unambiguously identify a conspecific solely by its vocalizations, called “individual vocal recognition”, allows for fast turn-taking communication in a crowd^1^. Individual vocal recognition is widespread in the animal kingdom and many mammalian and avian species are able to vocally recognize their partners, kin or group members^2–6^. Previous research on this topic shares a common feature: only the most prominent or common vocalization types of each species were examined. However, several species are known to produce a large set of different acoustic signals, allowing individuals to flexibly convey information in various contexts using a large vocal repertoire. Therefore, to understand the communication processes in a given species the whole of its vocal repertoire must be explored.

Zebra finches are group living, socially monogamous, highly vocal birds^7^. In the wild, in non-breeding condition, they usually form small groups of which the pair is the central unit^8^. In addition to the male song, wild zebra finches use up to eleven distinct call types^7^, a repertoire which can also be observed in captive birds^9–12^. Zebra finches are able to coordinate their responses to different call types within fractions of a second^11^. However, it remains unknown how such fast communication is achieved within a group. Specifically, it is not known whether birds can rely on auditory stimuli alone or whether they need other cues to recognize a caller. In a social situation it is predominantly the partner that answers most call types (produced by the mate)^11^ but previous research has only tested the males’ song, distance calls of adults and begging calls of chicks for recognition of individual identity^13–17^, without clarifying how soft calls are responded to in large groups. Zebra finch songs^13^ and distance calls^7, 16, 18^ can indeed be individually recognized. In playback experiments comparing distance calls of the mate and a familiar individual, adult males and females showed a different vocal response towards of the individuals depending on their familiarity^14, 15^. Building on this playback approach, we tested whether six types of zebra finch vocalizations contained information regarding individual identity: four different unlearned soft calls™stack, kackle, tet, and hat), the distance call and male song. The function of the soft calls is currently being refined, with the tet and stack are social calls often used for within pair communication^10–12^, the kackle is a breeding call used at the nest^19^, and the function of the hat call is not yet clear, but it is probably an alarm call previously named tuck or thuk^7, 12^. In our experiment, test subjects were presented with playback of three opposite-sex birds differing in their familiarity: (1) their partner, (2) a familiar and (3) an unfamiliar individual. The experiment was replicated on 2 successive days to establish the repeatability of birds’ responses. We predicted that if birds recognized individuals vocally, they would answer differentially based on the level of familiarity. We compared the type, number and latency of response to playbacks. Wireless backpack microphones^20^ allowed us to unambiguously record single birds during the experiment and during couples’ normal calling interactions prior to experimentation. Pairs are known to differ in the quality of their bond (fitness)^21^ and calling patterns^10, 22^, and these characteristics might be related^11^. Therefore, we also examined whether the strength of the relationship before the experiment had an influence on the number of answers during the playback experiment. Finally, we explored whether differences in spectral features of the playback stimuli influenced the observed response. If soft calls could be used to recognize individuals, they would allow birds to address specific individuals in vocalizing flocks.

## Results

### Call rate throughout the experiment

First we checked whether the birds habituated to our playback design. We found no consistent decrease in the vocal activity during the experiment (see Supplementary Fig. S1). There were no systematic variations observed in the number of calls; birds continued to use a similar rate of calls throughout the experiment. There was no statistical difference in the number of calls of the first and the last bin for males (p = 0.48) and females (p = 0.10) suggesting no habituation to the experimental design.

### Responses to playback: latency

Playback is an established method to test whether animal vocalizations contain individual identity, and we expected that different levels of familiarity between caller and receiver elicit different vocal responses.

**Figure 1:**
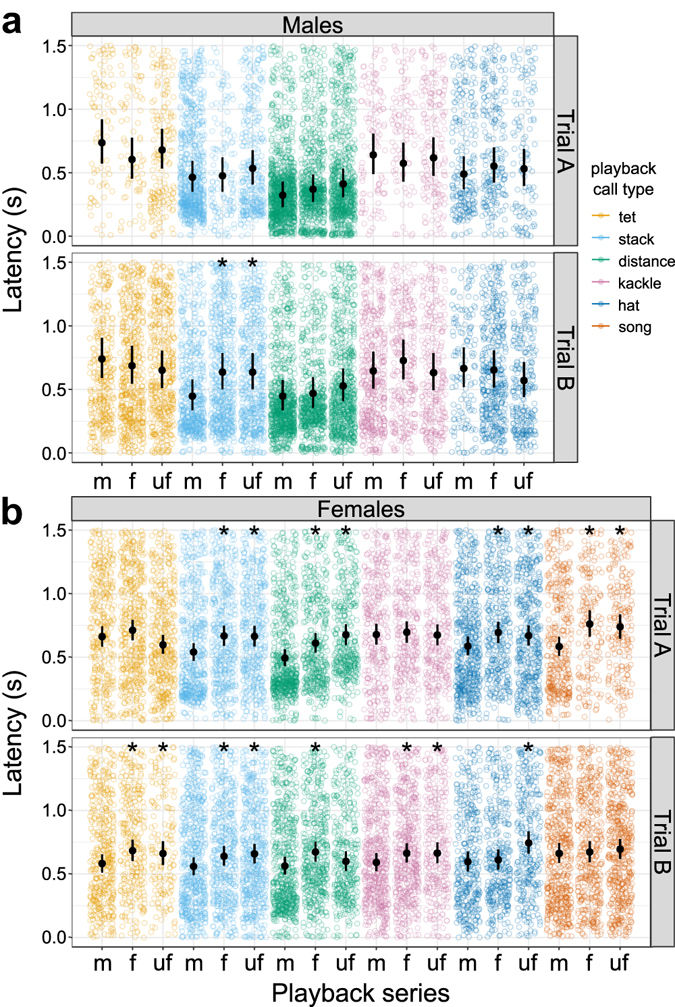
Latency during playback. Latency to the first answering call for different playback series (analysed time interval: 0–1.5 s after the onset of the playback stimulus). Colours represent the type of playback call broadcast; dots indicate individual calls (raw data). Familiarity categories: m = mate of the focal bird; f = familiar individual; uf = unfamiliar individual. For males (**a**) and females (**b**) in both trials the computed 95% credible intervals (error bars) as well as the fitted value (black symbols) are shown. Significant differences between mate and the other familiarity categories are marked by black asterisks.

First, we examined whether the latency of response to the playback calls differed by familiarity. In males we found a single significant difference (Fig. 1a, see Supplementary Table S1): individuals answered faster to stack calls of their mate than to those of non-mates during the second trial (trial B, mean ± SD, mate: 0.52 s ± 0.37 s, familiar: 0.65 s ± 0.42 s, unfamiliar: 0.64 s ± 0.41 s; differences between fitted values, i.e. effect size: stack-m – stack-f 0.19 s faster, p = 0.0018; stack-m – stack-uf 0.19 s faster, p = 0.0018). The difference between the two trials was due to a slower response to both familiar and unfamiliar calls in trial A (compare: trial A, familiar: 0.55 s ± 0.39 s, unfamiliar: 0.59 ± 0.41); in contrast, the latency to respond to the mate’s playback remained similar in both trials (trial A, mate: 0.55 s ± 0.39 s).

For all female call types we found at least one statistically significant difference for one of the trials (Fig. 1b, see Supplementary Table S2). When answering stack calls, females responded faster to their mate than to familiar and unfamiliar birds in both trials (mean ± SD, mate: 0.59 ± 0.40 (A), 0.65 ± 0.37 (B); familiar: 0.70 ± 0.39 (A), 0.69 ± 0.39 (B); unfamiliar: 0.72 ± 0.39 (A), 0.67 ± 0.38 (B); effect size for each trial, stack-m – stack-f p = 0.0004 (A), p = 0.0142 (B); stack-m – stack-uf = p = 0.0005 (A), p = 0.0036 (B)).

Results regarding the song and remaining four calls were equivocal between trials. However, all statistically significant differences in each trial always went towards the expected direction: the focal individual responded faster to the partner than to a familiar or unfamiliar male.

For distance calls, answers to the mates were always faster than to those of familiar and unfamiliar males in both trials (mean ± SD, mate: 0.51 ± 0.35 (A), 0.58 ± 0.38 (B); familiar: 0.64 ± 0.37 (A), 0.68 ± 0.37 (B); unfamiliar: 0.69 ± 0.35 (A), 0.64 ± 0.36 (B)). The latencies to unfamiliar calls in the second trial were the only ones that were not significantly slower than the answers to a mate’s calls (effect size, distance-m – distance-f = 0.12 s, p = 0.0004 (A), 0.11 s, p < 0.0001 (B); distance-m – distance-uf = 0.18 s, p <= 0.016 (A), 0.04 s, p = 0.16 (B)).

For hat calls, the answers to the mate were also faster than those to familiar and unfamiliar individuals (mean ± SD, mate: 0.62 ± 0.39 (A), 0.63 ± 0.39 (B); familiar: 0.76 ± 0.40 (A), 0.65 ± 0.38 (B); unfamiliar: 0.71 ± 0.41 (A), 0.76 ± 0.41 (B)). All but one comparison were statistically significant (effect size, hat-m – hat-f = 0.11 s, p = 0.0032 (A), 0.01 s, p = 0.3497 (B); hat-m – hat-uf = 0.08 s, p = 0.0187 (A), 0.15 s, p < 0.0001 (B)).

With kackle calls, responses to the mate were significantly faster than to both familiar and unfamiliar calls only during the second trial (mean ± SD, mate: 0.75 ± 0.41 (A), 0.67 ± 0.40 (B); familiar: 0.75 ± 0.39 (A), 0.70 ± 0.38 (B); unfamiliar: 0.74 ± 0.40 (A), 0.74 ± 0.40 (B); effect size, kackle-m – kackle-f = 0.02 s, p = 0.3322 (A), 0.07 s, p = 0.0256 (B); kackle-m – kackle-uf = 0 s, p = 0.5333 (A), 0.07 s, p = 0.0235 (B)).

The answers to songs of the mate were also significantly faster than those to familiar and unfamiliar songs for the first but not for the second trial, despite trends in the expected direction (mean ± SD, mate: 0.66 ± 0.41 (A), 0.68 ± 0.42 (B); familiar: 0.79 ± 0.41 (A), 0.70 ± 0.42 (B); unfamiliar: 0.78 ± 0.38 (A), 0.71 ± 0.40 (B); effect size, song-m – song-f = 0.18 s, p < 0.0001 (A), 0.01 s, p = 0.3855 (B); song-m – song-uf = 0.16, s p = 0.0004 (A), 0.03 s, p = 0.2033 (B)).

For tet calls, there was a significantly faster response to the mate compared with the other two levels of familiarity during trial B (mean ± SD, mate: 0.76 ± 0.41 (A), 0.67 ± 0.40 (B); familiar: 0.75 ± 0.41 (A), 0.77 ± 0.39 (B); unfamiliar: 0.65 ± 0.39 (A), 0.75 ± 0.41 (B); effect size, tet-m – tet-f = 0.05 s, p = 0.116 (A), 0.10 s, p = 0.0048 (B); tet-m – tet-uf = −0.06 s, p = 0.9474 (A), 0.08 s, p = 0.0196 (B)).

Taken together, our results demonstrate that females can identify individual identity upon hearing males’ stack calls. Although inconclusive, our results suggest that partners can be distinguished from individuals of differing familiarity also by the remaining call types.

### Response to playback: number of calls per playback series

Second, we considered the total number of calls emitted during each playback series. Overall, the number of answers did not differ between the two trials (probability trial B > trial A; p = 0.60 for females and p = 0.75 for males).

**Figure 2:**
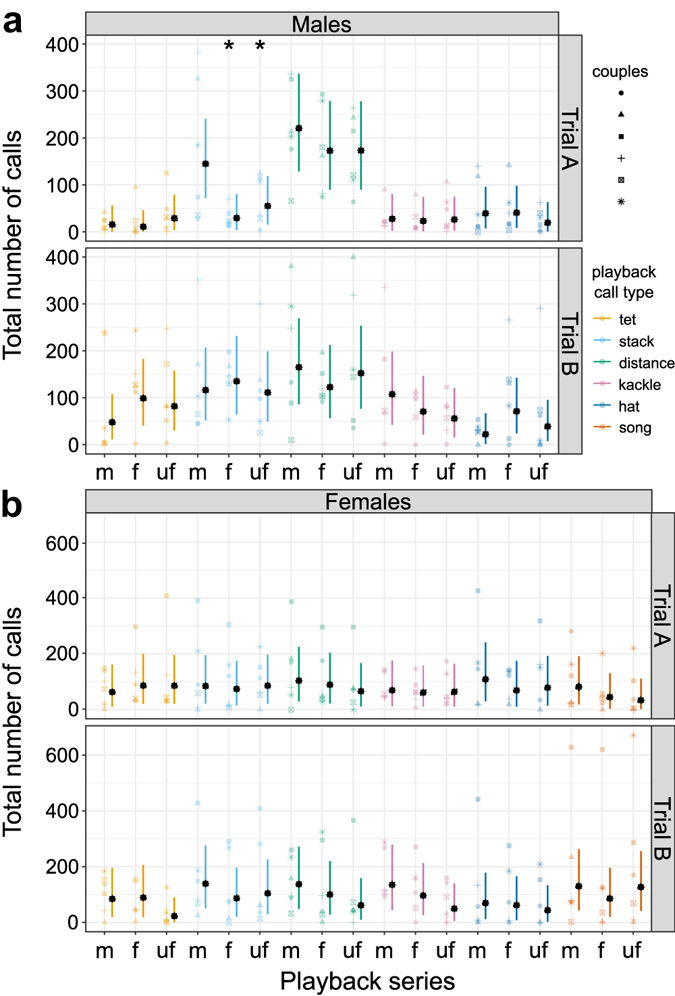
Number of calls during playback. Number of answering calls that focal individuals emitted during the different playback series. Raw data for males (**a**) and females (**a**) and both trials (symbols indicate responses of individual birds) and the computed 95% credible intervals (error bars) as well as the fitted value (black symbols) are shown. Colours represent the type of playback call broadcast. Familiarity categories are indicated by letters: m = mate of the focal bird; f = familiar individual; uf = unfamiliar individual. Significant differences between mate and the other familiarity categories are marked by black asterisks.

In males, during trial A, the number of answers elicited by stack calls depended strongly on their familiarity. Male zebra finches responded with a higher number of calls to the stack calls of their pair-bonded females (mean ± SD of number of calls, 171.8 ± 152.9) than to those of a familiar (29.7 ± 22.1; probability stack-m < stack-f = 0.0003) or unfamiliar female (69 ± 52.6, probability stack- m < stack- uf = 0.0064; Fig. 2a). No other comparison reached statistical significance in males. In females, the number of answers showed no strong relationship with the level of familiarity of the caller (Fig. 2b). These results suggest that males can identify individual identity by hearing females’ stack calls.

### Type of answering call

Males and females responded to playbacks using different call types. Females predominantly responded with stack calls (69.2% of the total answers), followed by distance calls (22.4%) (see Supplementary Fig. S2). In males we found a similar pattern, the majority of their responses being stack call (50.1%), followed by distance calls (17.6%) and hat calls (11.8%) (see Supplementary Fig. S3). Not only the number of calls or their latency, but also the quality of the answer (type of call used to answer) might differ when responding to different familiarity levels. However, we did not find any such differences (see Supplementary Tables S3 and S4 for females and males, respectively). Thus, we conclude that the level of familiarity did not influence the type of response to playback.

### Habituation within playback series

Because the specificity of the answers for the different familiarity levels could change during the experiment due to behavioural habituation, we compared results obtained during the first and last 30 playback calls of each series with those of the complete series (300 calls). Regarding the number of calls, in males, we found only two statistical differences between the two datasets for both first and last playback calls (see Supplementary Figs S4a and S5a) which were in the expected direction, there were more calls in response to the mate compared with the other familiarity levels. In females, we also found only three differences between response to the first 30 playback calls versus the complete dataset, and only one difference between response to the last 30 playback calls and the complete dataset (see Supplementary Figs S4b and S5b). Thus also for females we found limited differences between datasets and all in the expected direction with more answers to mates. Interestingly, considering only the first playback calls in the first trial we found more answers to mates compared to both of the other levels of familiarity when answering male songs.

There were very few differences in response to experimental conditions over time when considering the latency of response to playback when comparing the full dataset to the first 30 calls (see Supplementary Fig. S6 and Tables S5 and S6) and to the last 30 calls (see Supplementary Fig. S7 and Tables S7 and S8). Only when considering the 30 call subsets, males showed significant differences in the direction opposite to our expectations in kackle and hat, i.e. slower answers to the mate than to non-pair-bonded females. This might be due to the rarity of elicited answers and the absence of a real pattern leading to false positive results.

For females, for which we showed that latency was very important (Fig. 1b), there was only one case out of 24 for each first and last 30 playback calls dataset where the direction of the difference opposed our expectations. The majority of differences between latency to respond to the mate versus other familiarities observed in the full dataset were also present in the subset data, especially in the first trial (A) (see Supplementary Figs S6b and S7b); a change in response to experimental treatment over time occurred during the last 30 playback calls of the second trial (B) were most of the differences with the full dataset are concentrated (see Supplementary Table 8). These results confirm that the quality of the answer did not change much throughout the experiment and that longer playback series produced more reliable results.

### Relation between calling behaviour during baseline and experiment

We investigated whether the calling relationship of mates before the experiment influenced the number of answers during the experiment. We first asked which call combinations showed repeatable patterns during the baseline period and, therefore, might result in a predictable answering rate during the playback experiment (see Supplementary Table S9). We therefore considered only the stack-stack exchanges whose percentage of answers was consistent during baseline recording for males (repeatability = 0.995 + 0.004) and females (repeatability = 0.83 + 0.13). Subsequently, we correlated the percentage of answers during the playback experiment (separately for trial A and B) with the percentage of answers during the baseline (mean of both days). There was no relationship between baseline and experiment in females (trial A p = 0.7905, trial B p = 0.4784) but we detected a negative relationship in males (trial A p = 0.0325, trial B p = 0.0523) (see Supplementary Fig. S8). The relationship between response during baseline and experimental periods in males demonstrates that vocal performance during the experiment is related to preceding vocal relationships.

### How the variability of call type spectral features is related to the variation in conspecific response

Finally, we identified the most individually distinct call types and whether the individual variability of each call type was a good predictor of the conspecific response (quantified as the number of calls and latency to respond). We aim to demonstrate that what we interpret as recognition is not a by-product of easier discrimination. According to our analysis the hat was the most distinct call type for both males and females. The stack call, although unambiguously recognised by both males and females, was not the most distinct call type (see Supplementary Fig. S9a). Furthermore, the magnitude of the response (response to the mate – response to the familiar/unfamiliar) was not correlated with the index of individual distinctiveness (see Supplementary Fig. S9b). Because the most individually distinct call types were not the ones recognised best, we demonstrated that there was recognition beyond discrimination.

## Discussion

Females and males unambiguously showed individual recognition of stack calls produced by the opposite sex. We found a differential response to distinct familiarity levels, both in the number and timing of the response. Females only used timing to demonstrate recognition: they vocalized at similar rates when responding to the playback of different familiarity levels, but responded more quickly to the calls of their mate versus non-mates. Intriguingly, males used multiple strategies in different trials to demonstrate recognition: a higher number of calls in response to mates during the first and a shorter latency to respond during the second. Furthermore, in females, consistent differences between answers to their mate and those to at least one other level of familiarity were detected for two call types (hat and distance call). Only latency and number of calls regardless of type differed between familiarity levels.

Until recently, soft calls have been considered “a background hum in which other calls are embedded (…), not directed at specific individuals and do not stimulate specific replies”^7^. In contrast to this view, growing evidence demonstrates that soft calls are indeed directed at individuals and can elicit specific replies^10, 11, 22^. In group contexts, addressing specific subjects is a prerequisite for effective communication and we now provide the missing link explaining how this is achieved: we show that soft calls can be assigned to individuals and that the latency of an answer can provide specific information. For stack calls, we estimated a difference between the answer to the mate and familiar or unfamiliar individuals of approximately 188 ms for males and 125 ms for females. This delay is roughly similar to the mean latency of calls used as replies and double the length of stack calls^10^; therefore, this response gap can be biologically relevant and directly used within a communicating group. Individual vocal recognition using contact calls has rarely been investigated in Passeriformes although different functions have been proposed: for example, Large-billed Crows (*Corvus macrorhynchos*) can recognize strangers’ loud calls^23^, Long-tailed bushtits (*Aegithalos caudatus*) kin’s contact calls^6^, Chestnut-crowned Babblers (*Pomatostomus ruficeps*) group members’ contact calls^24^ and Silvereye (*Zosterops lateralis*) mates’ calls^25^. However, in most of the studies it was unclear whether these calls were learned and a large proportion of the typical repertoire remained untested. Therefore, more research is needed to identify common themes in the evolution of recognition of soft vocalizations and to establish whether addressing specific individuals in a vocalizing group is common among Passeriformes.

Females tended to respond differently to their partner’s vocalizations in most call categories tested, whereas males’ answers only differed when responding to stack calls. We cannot yet explain this result, but differential discrimination abilities^26^ and sex-specific roles in the communication process have been proposed^27^. Despite several lines of evidence indicating that females might be able to recognize individual identity from all soft call types, we could only confirm this for hat and stack calls. This may be due to the differing functions of specific call types. For example, recognition of the hat call might be important in identifying the alarming bird, a colony member or an external individual, whereas recognizing the stack call may serve to maintain vocal contact with a mate. Tet and kackle calls, in contrast, are part of the private communication occurring at the nest where other individuals are not present and thus, individual recognition may be less important^12, 19^. Breeding calls, such as the kackle, become more common once a couple is nesting^11^. The stack call, on the other hand, is one of the most common call types in non-breeding groups^10, 11^, a situation which resembles the context of our playback experiment, which might explain why stack calls were promptly recognized in our study.

Notably, our results entail a process of comprehension learning of the stack call, which is important because comprehension learning is a prerequisite for the evolutionary origin of vocal learning^10, 28^. Soft calls are generally used when partners are in close proximity of each other – and therefore see each other^7, 12, 19^; hence cues other than acoustic modalities are available to facilitate individual recognition. Therefore, identifying individuals is indeed not the sole intention of these vocalization types; the encoded identity can be used in communication between specific individuals in a group. Our results suggest that birds are integrating information about call type and call identity to tailor vocalizations and provide the correct answer type and time. Previous observations and multiple independent lines of evidence led us to postulate that our findings agree with the hypothesis that vocal learning is driven by social complexity^29^. Learning acoustic parameters is a precursor for any subsequent learned modification of the spectral features of a vocalization^30^. Moreover, soft calls are encoded in a high order telencephalic nucleus of the motor pathway^10^, which is fundamental for the control of learned vocalizations, and may facilitate coordination of communication^31, 32^. Therefore, although vocal recognition is present in many vocal non-learners^5^ and comprehension learning may just be a prerequisite rather than a driving force, we suggest that unlearned calls in vocal learners might provide a model to better understand the origin of vocal learning capabilities.

Until now, only part of the repertoire of the zebra finch had been tested for individual recognition. As for many other Passeriformes^33^, song has repeatedly been shown to contain individual characteristics that can be used for identification^7, 27, 34^. In most cases this has been proven in simultaneous choice tests^13, 35, 36^. In contrast, higher vocal response towards the partner’s song was only reported once and exclusively when vocalizations emitted towards the speaker were taken into account^36^. Our results regarding song are equivocal; females showed a differential response during their first trial, confirming previously published results, but this response did not hold during the second trial, which could partially be explained by habituation to the experimental design for this vocalization type. In addition to song, several studies investigated the distance call of both sexes, attempting to assess whether these vocalizations contain individual information and whether this information is used for identification^13–15, 37^. In our experiment, we found sex-dependent behavioural responses to recordings of distance calls from individuals of different familiarity. Female zebra finches displayed more and faster responses towards their mate’s calls than towards familiar and unfamiliar distance calls, thereby confirming previously reported discrimination capabilities^14^. Conversely, in males, no such differences were found, despite trends in the expected direction. Our results thus diverge from previous studies, which demonstrated a significantly higher number of answers during the replay of the mate’s than of familiar distance calls. However, in previous studies only distance calls were regarded as answers^15^ or only considered the neuronal response in males’ high-order auditory areas (Caudomedial Mesopallium, NCM)^38^. Furthermore, the difference between our and previous studies might be due to differences in the selection method for playback stimuli. We extracted distance calls from the normal communication flow, i.e. calls uttered when both partners remained in the same sound-proof box. In contrast, previous studies used provoked distance calls elicited by visually separating the individuals^14–17, 39, 40^. Because social context can influence the acoustic structure of a bird’s call^40^, it is possible that provoked calls emitted by birds in isolation exhibit enhanced call urgency in order to initiate contact with their partner. This call urgency might in turn increase the motivation of focal subjects to respond to these calls in a playback setup, possibly explaining why males in previous experiments showed a higher vocal response to their partner’s calls than in our study.

We used natural rather than synthetic vocalizations for playback to ensure that stimuli contained all necessary acoustic structures, as altered call perception is possible when using artificially created vocalizations^41, 42^. Additionally, we employed long playback sessions to increase power for our analyses. Most playback studies use very few calls to avoid habituation. Instead, we attempted to mitigate habituation by continuously varying the interval between successive calls. Despite the long duration, calling rate did not decrease during the experiment or during the single series, indicating that the birds did not habituate to the playback. Additionally, differences between the results obtained for the first 30 playback calls compared to those for all 300 calls were negligible. When comparing the last 30 playback calls to the entire dataset the only noteworthy differences occurred during females’ second trials which may indicate a certain degree of habituation. This is remarkable because we found that low numbers of answers (e.g. familiar kackle and hat in males, see Supplementary Figs S6a and S7a) actually increased improbable and false positive results. We repeated the entire experiment on two consecutive days to assess whether birds habituated to the playback design. Indeed we found differences between trials, but not concerning the stack call, which was always answered differentially according to familiarity level. The differences between trials are difficult to interpret and should be considered when planning experiments that contain multiple presentations of the same stimulus. Finally, we did not observe an effect of social context required for answer specificity^14, 15^, the audience pairs, as their calls did not influence the results directly. Specifically, backpack microphones worn by the focal birds assured the individuality of the recordings^20^, and the effect of the audience, quantified in our models, was limited.

Stress is also a possible confounding factor in our experimental setup. Notably, corticosterone levels in zebra finches increase 24 hrs after the separation of an established pair^43^. Moreover, these hormones are associated with a reduction in vocal discrimination ability^40^. Therefore, mate separation before and during the playback experiment might have increased stress levels in the test subjects, thereby impairing their discrimination abilities. Although we endeavoured to reduce the stress for focal birds by limiting the separation period to one night before the first experimental trial, the different personalities of the test subjects may have led to differences in stress response^44^. In addition, the quality of the pair bond itself might influence the level of stress birds experience when separated from their mate; couples sharing a stronger bond might be more strongly affected by separation than those having weaker pair bonds. This might partially explain why in males, which are highly repeatable in their response^22^, we found a negative relationship between the proportion of answers during playback experiments and the baseline. Unfortunately, the small sample size of our study makes it difficult to generalize these findings; however, the correlation with measurements of pair strength is worth further investigation.

Vocal individual recognition of the so-called “soft calls” has not previously been tested in zebra finches; we provide the first evidence that at least one of these call types, the stack call, contains individual identity despite not being more individually distinct than other soft calls. This finding implies that soft unlearned acoustic signals are sufficient to determine a caller’s identity and that visual cues are not required. We have identified the mechanisms underlying how birds vocally interact in a group. Namely, employing differential latency times when answering to different subjects allows a caller to address individuals specifically. Vocal recognition is a fascinating aspect of vocal communication because the ability to recognize individuals in a group of vocalizing conspecifics is a prerequisite for complex communication networks.

## Material and Methods

### Ethics statement

The use of audio transmitters, bird housing in sound-proof boxes, and conduct of all other experimental procedures were approved by the government of Upper Bavaria (record number 55.2-1-54-2532-21-2015). All further animal husbandry and handling was conducted according to the directives 2010/63/EU of the European parliament and of the council of 22 September 2010 on the protection of animals used for scientific purposes.

### Animals and housing conditions

A total of 12 adult zebra finches, six pairs, served as the focal birds for the experiment, plus 14 additional birds which served as the audience (i.e. as company for focal birds)^15^. All pairings were “forced”, i.e. couples were formed by randomly selecting unrelated individuals from the breeding facilities of the Max-Planck-Institute for Ornithology, Seewiesen, Germany. Birds were kept in a 13/11 Light/Dark cycle, at 24 °C and 60–70% humidity. Food (mixed seeds, and “egg food”), fresh water and cuttlebone were provided *ad libitum*. We performed all experiments with birds from forced pairs in the non-breeding condition. All couples had been together for at least six months, raised at least one brood and had been housed without nesting material for three months prior to the experiment. Zebra finch couples were housed in single pair-cages (123.0 cm × 37.0 cm × 38.5 cm) in two separate rooms with three experimental couples per room. Within each room couples could see and hear each other, whereas there was no acoustic or visual contact between pairs housed in different rooms, making these two groups “unfamiliar” to each other. Experimental pairs were housed with other breeding pairs, seven of which served as “audience couples” during the playback experiments.

### Experimental timeline, sound recording and playback

Zebra finch couples were moved to sound-proof boxes one week before the experiment to allow for acclimatization to the new conditions. Animals were equipped with custom-made light-weight (less than 5% of average body weight) wireless microphone transmitters fitted on their back via a leg-loop harness as previously described^20^ which recorded continuously throughout the experiment. To determine the vocal relationship between males and females^10, 22^, we audio recorded each pair for three consecutive days, using the recordings of the first and third day as the baseline for subsequent analysis of calling patterns. Audio was scored for four hours a day (12:00 to 16:00). Each sound-proof wooden box was equipped with a general microphone (TC20; Earthworks, USA) which was used to extract playback stimuli.

To create the familiarity level “familiar” (equivalent to a group member in the wild), we moved a couple from the same housing facility as the focal pair into their cage during the evening of day 3 (end of baseline recording). The two pairs shared a cage for approximately 24 h, separated by a wire mesh allowing acoustic and visual interaction. Their calls served as “familiar stimuli” during the playback experiments^37, 38^. During the night of day 4, the “familiar” couple was removed and the male and female of the focal couple were separated. Each focal bird was placed with an unfamiliar, established pair: the “audience couple”. This audience provides a social context that increases the specificity of the answers^14, 15^ and prevents social isolation^40^. Experiments were carried out during the morning and afternoon of days 5 and 6, resulting in one trial per bird per day. The time of testing (morning/afternoon) was randomized, and audience couples were changed between trials (i.e. in the evening of day 5).

The replay of calls was controlled via a computer connected to an amplifier (CS-PA1, SINTRON Vertriebs GmbH, Germany), and calls were broadcast via a loudspeaker (KFC-1761S, Kenwood Electronics, UK) placed at the back wall of the sound-proof box. The sound level of the experimental signals, measured at a distance of 1 m from the loud speaker (Sound Meter, Model HD600, Extech Instruments, U.S.A), was adjusted to a peak value between 50.03 dB ± 0.87 dB (mean ± SD; minimum for the lowest call type, the tet) and 74.05 dB ± 1.15 dB (corresponding to the loudest call type, the distance call reflecting a typical level of a natural distance call)^39^, and was constant for all three familiarity levels of each call type.

The playback stimuli and the focal bird’s calls were recorded (via external and backpack microphone, respectively) synchronously on separate audio channels for subsequent alignment. Each subject was presented with calls of three different individuals of the opposite sex, representing three familiarity levels: “mate” (m; partner of the focal individual), “familiar” (f; known individual), and “unfamiliar” (uf; unknown individual). Six different vocalization types were used for playback experiments: tet, stack, distance call, kackle, hat, and - in the case of females - song (for the original audio files see additional information). Playback calls were extracted from the general microphone recordings during the acclimatization phase and baseline period. In rare cases in which birds did not emit a specific call type during the sampling period, this type was omitted from the playback. Each vocalization was high-pass filtered (freq = 85 Hz), its amplitude normalized to 0.1 dB (maximal sample value) and the stimulus faded in and out to avoid rapid amplitude changes. Playback calls were presented in blocks, each block consisting of three series of playback calls of the same call type. Each series consisted of calls of an individual representing one of the three different familiarity categories (m, f, and uf). We used three randomly selected vocalizations of each call type per individual in order to mitigate pseudo-replication^45^. Within a series, each playback call was repeated 100 times for a total of 300 calls. To ensure that the playback was unpredictable, the inter-call intervals were changed randomly at each emission (within 2 ± 0.5 s, uniform distribution). Playback series within a block were interspaced by 70 ± 10 s of silence, and different call-type blocks by 130 ± 10 s of silence. The total duration of an experimental trial was approximately 2:45 h for males (15 series) and 3:30 h for females (18 series). The order of call-type blocks and of familiarity categories within blocks, as well as the order of single calls within series, were determined semi-randomly.

### Sound analyses and sorting of vocalizations

Sorting of vocalizations from audio files proceeded as previously described^10, 20^. Briefly, sounds that exceeded a manually set amplitude threshold were extracted for further analysis. Using custom software written in Delphi Pascal for Windows (SoundExplorer; R. F. Jansen, MPIO, 2000; see ref. 11 for GitHub address), the following parameters of each sound were computed: average frequency, modal frequency, fundamental frequency (first peak), Wiener entropy, duration, and their standard deviations. The subsequent clustering process was based on a k-means clustering algorithm^46^. After noise detection and elimination, the results were refined manually: each cluster was checked and sorting errors were corrected, resulting in a separate cluster for each call type of a bird’s repertoire^10, 11^. Information about the call type and the timestamp was saved for each vocalization. We used this information to determine the temporal relationship between all possible call type combinations of mates during the baseline^10, 11^ and during the playback experiments. During the baseline period we considered all calls emitted within 0.5 seconds from the partner’s call as answers. For each combination of call types, we calculated the number of answers and the proportion of answers from the total amount of emitted calls of that specific type (see ref. 22 for details on calculations).

### How the variability of call type spectral features is related to the variation in conspecific response

We then explored whether the most individually distinct call types were the ones that were easier to discriminate. We extracted 14 acoustic features^12^ of each call sonogram tested as playback stimuli (i.e. for each call type three calls for individual of each familiarity). which were then used to conduct principal component analysis (PCA)^47^ for each call type. We extracted the mean frequency and its standard deviation, median frequency, skew, kurtosis, spectral flatness measure, entropy, mode frequency, frequency precision of the spectrum, peak frequency, fundamental frequency, dominant frequency, maximum dominant frequency, and duration (R package “seewave”)^48^. Subsequently, we used the first two principal components as explanatory factors in a linear discriminant analysis (R function “lda”)^49^ with familiarity level as a predictor. We used the parameters obtained to predict (R function “predict”)^49^ the proportion of cases in which the calls were assigned to the correct familiarity. We ran this analysis for each focal bird and we present the average and SD of the incorrect assignments (see Supplementary Fig. S9). Call types in which the individuals are very distinct will have a lower proportion of incorrectly assigned calls. Lastly, we used the within call type variability as a predictor of the magnitude of the response to the conspecific (number of calls and latency, response to the mate – response to the familiar/unfamiliar – for the latency the average for each individual was taken) in a linear mixed model with random factors as described below.

### Statistics

All statistical analyses were performed using R^50^ in a Bayesian framework. We used linear mixed-effect models^51^ to analyse the effect of different playback stimuli on the total number of response calls emitted during each playback series, and on the latency of the first answering call for each playback series (upper limit 1.5 s) separately by sex. Before interpreting the results, we checked whether model assumptions were met by inspecting the residuals for normality, homoscedasticity, and lack of remaining pattern. Both the total number of responses and latency were square root transformed to approximate normality. Three categorical variables served as fixed effects in both models: familiarity (3 levels: mate, familiar, and unfamiliar), playback call type (5 levels: distance, stack, kackle, hat, and tet; 6^th^ level for females: song) and trial (2 levels: trial A and trial B), as well as all interactions. We included individual identity (12 levels), audience (i.e. the identity of the audience pair, 7 levels) and playback order (i.e. the order in which playback series were broadcast, 15–18 levels) as random factors. Model structure was based on the study design rather than model selection. Familiarity was expected to influence both outcome variables (total number of response calls and latency of the first answering call). Playback type and trial were also hypothesized to affect the outcome differentially by familiarity in the distinct playback call types. Therefore the interactions of all these variables were included. In order to obtain parameter estimates we used Maximum Likelihood (ML) because we were most interested in fixed effects^52^. We calculated credible intervals (CrI) using the function “sim” from the R package “arm”^53^. A total of 10000 values were simulated from the joint posterior distribution of the model parameters. If the CrI of different playback categories did not overlap, the results were considered significantly different from each other. In cases where CrIs overlapped, but the fitted values differed largely between playback series, a derived calculation from the aforementioned simulated values was performed. For this purpose, simulated values of the two groups of interest were compared (10000 comparisons), and we reported the number of cases in which the value of the first group was larger than the one of the second group. If this condition held true for less than 5% of the cases, the mean response of the first group was regarded as significantly smaller than that of the second.

To determine whether birds had habituated to the playback experiment, we investigated changes in calling rate throughout the experiment. First, we counted the events occurring in 500 s bins (roughly the length of a playback series) for each bird and trial. We then performed a linear mixed model with the number of calls as the dependent variable and bins (18 levels for males, 21 for females) as the explanatory variable with individual ID as a random factor. The parameters were simulated 10000 times to estimate fitted values and CrI from the outcome of the model. This allowed an assessment of patterns and enabled us to test whether there was a statistical difference in the number of calls between the first and the last bins.

We tested whether the familiarity of the playback affected the type of answer. For each trial we scored and counted the number of calls within 0.5 s of every playback call. We then calculated the proportion of each type of answer out of the total answers for each bird. Because very low counts might easily influence the proportions, we set a threshold of 5 answers to each playback series in order to be considered (dataset included as additional information, see Supplementary Table 3). For each playback series we compared the proportion of call types of the three familiarities using a non-parametric test (Kruskal-Wallis rank sum test). We ran the test only when there were at least 8 non-null values per series (i.e. the sample size was at least 8 times the number of explanatory variables)^54^.

Additionally, we investigated whether we could explain individuals’ answering rate by the pair’s vocal relationship established before the experiment (during “baseline”). Among all call combinations used during “baseline”, we selected only those in which each bird had used at least 3 calls in order to rule out inconsistent and rare combinations. For the resulting combinations we calculated a repeatability index^22^ because only in case of a repeatable behaviour we can expect a consistent response during the experiment. We calculated repeatability according to the F ratio: the mean squares among groups divided by the mean squares within groups^55^. Finally, for repeatable call combinations, we quantified the correlation between the proportion of answers (i.e. of calls emitted within 0.5 s) during playback and baseline.

### Data availability

Data available from the Dryad Digital Repository: D’Amelio B., P., Klumb., M., Adreani, N. M., Gahr, L. M. & ter Maat, A. Data from: Individual recognition of opposite sex vocalizations in the zebra finch. Dryad Digital Repository. doi:10.5061/dryad.4g8b7 (2017).

## Electronic supplementary material

Supplementary Figures and Tables
