# Supplementary material for: Individual recognition of opposite sex vocalizations in the zebra finch
Source: Sci Rep. 2017 Jul 17;7:5579. doi: 10.1038/s41598-017-05982-x (PMC5514125; doi:10.1038/s41598-017-05982-x)
Supplement: Supplementary file 1 — Supplementary Figures and Tables [file 41598_2017_5982_MOESM1_ESM.pdf]

Supplementary Materials for

Individual recognition of opposite sex vocalizations in the zebra finch

Pietro B. D'Amelio*, Milena Klumb, Mauricio N. Adreani, Manfred L. Gahr, and Andries ter Maat

*Correspondence to: pdamelio@orn.mpg.de; pie.damelio@gmail.com

This file includes:

Supplementary Figures S1 to S9 and Table S1 to S9

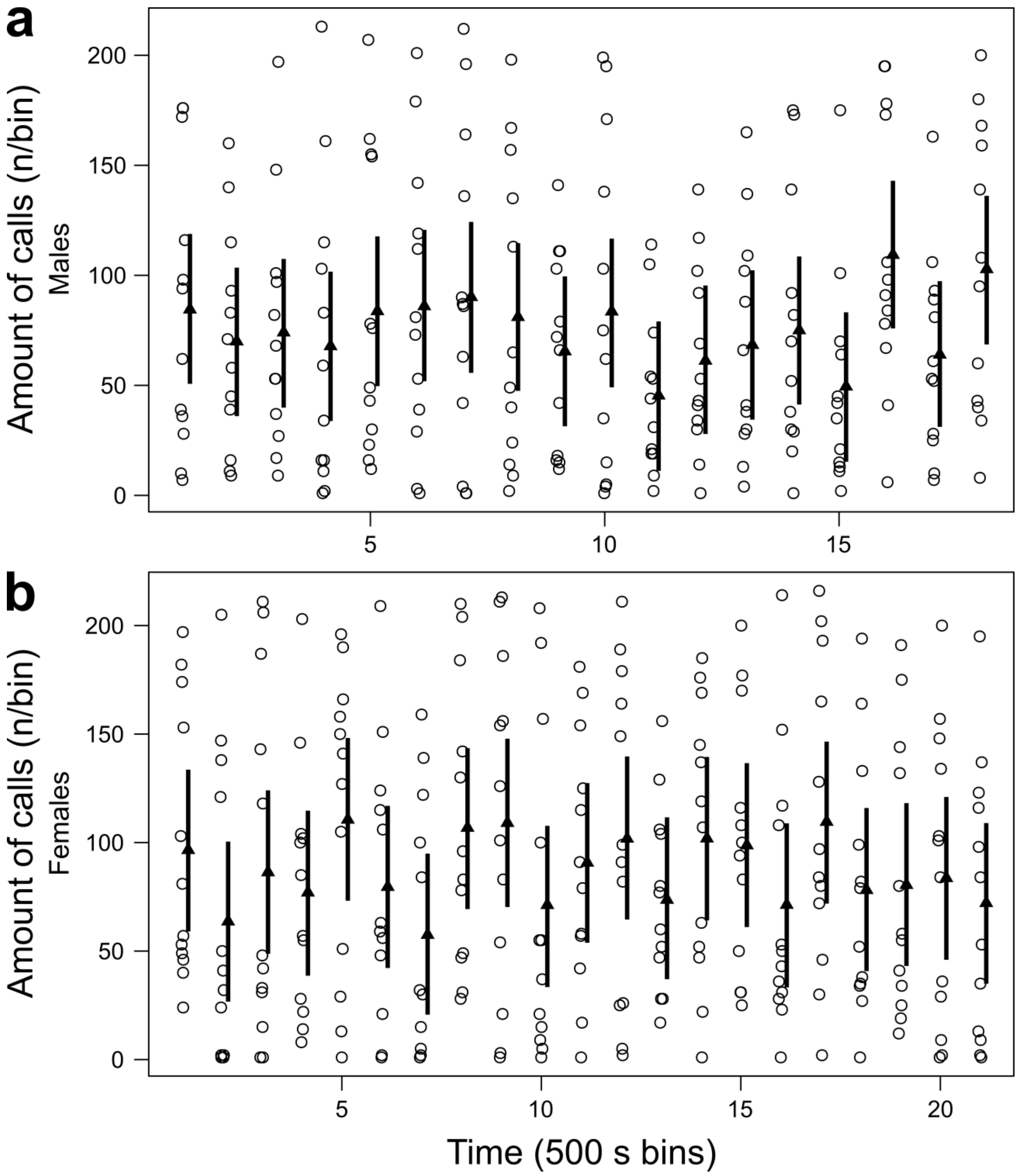

Supplementary fig.1

Acclimatization of the number of call. Number of calls focal individuals emitted during the entire playback experiment, divided in 500 s bins. Shown are the raw data of both trials for all 12 individuals, divided in males (top, a) and females (bottom, b). Dots represent the sum of responses each individual emitted per 500 s bin by trials; error bars indicate estimated credible intervals and fitted values from the linear mixed model. We notice that in contrast to what we would expect in case of becoming accustomed to the playback stimuli; there is not a steady decline of the number of calls during the playback. There is no statistical difference between the first and the last bin.

Call type	Familiarity	Latency		P. mate slower	
		**A**	**B**	**A**	**B**
	Mate	0.89 ± 0.45	0.63 ± 0.40		
Tet	Familiar	0.80 ± 0.44	0.66 ± 0.42	0.9419	0.755
	Unfamiliar	0.66 ± 0.42	0.68 ± 0.42	0.743	0.8757
	Mate	0.55 ± 0.38	0.52 ± 0.37		
Stack	Familiar	0.55 ± 0.39	0.65 ± 0.42	0.4206	**0.0018**
	Unfamiliar	0.60 ± 0.41	0.64 ± 0.41	0.1343	**0.0018**
	Mate	0.39 ± 0.30	0.43 ± 0.32		
Distance	Familiar	0.44 ± 0.34	0.45 ± 0.33	0.1836	0.3585
	Unfamiliar	0.48 ± 0.38	0.53 ± 0.38	0.0549	0.0957
	Mate	0.76 ± 0.41	0.64 ± 0.41		
Kackle	Familiar	0.83 ± 0.38	0.72 ± 0.41	0.7985	0.1429
	Unfamiliar	0.76 ± 0.41	0.64 ± 0.42	0.6141	0.571
	Mate	0.63 ± 0.42	0.72 ± 0.43		
Hat	Familiar	0.71 ± 0.42	0.64 ± 0.40	0.1844	0.5638
	Unfamiliar	0.69 ± 0.48	0.62 ± 0.42	0.2669	0.889

Supplementary table 1

Latency of males' calls to females' playback. The mean ± the standard deviation of each playback series is reported together with the probabilities (P) that calls emitted during mate series have a longer latency than calls emitted during the other familiarity categories. The statistical significant differences are reported in bold.

Call type	Familiarity	Latency		P mate slower	
		**A**	**B**	**A**	**B**
Tet	Mate	0.76 ± 0.41	0.67 ± 0.40		
	Familiar	0.75 ± 0.41	0.77 ± 0.39	0.116	**0.0048**
	Unfamiliar	0.65 ± 0.39	0.75 ± 0.41	0.9474	**0.0196**
Stack	Mate	0.59 ± 0.40	0.65 ± 0.37		
	Familiar	0.70 ± 0.39	0.69 ± 0.39	**0.0004**	**0.0142**
	Unfamiliar	0.72 ± 0.39	0.67 ± 0.38	**0.0005**	**0.0036**
Distance	Mate	0.51 ± 0.35	0.58 ± 0.38		
	Familiar	0.64 ± 0.37	0.68 ± 0.37	**0.0004**	**0.0001**
	Unfamiliar	0.69 ± 0.35	0.64 ± 0.36	**0.016**	0.16
Kackle	Mate	0.75 ± 0.41	0.67 ± 0.40		
	Familiar	0.75 ± 0.39	0.70 ± 0.38	0.3322	**0.0256**
	Unfamiliar	0.74 ± 0.40	0.74 ± 0.40	0.5333	**0.0235**
Hat	Mate	0.62 ± 0.39	0.63 ± 0.39		
	Familiar	0.76 ± 0.40	0.65 ± 0.38	**0.0032**	0.3497
	Unfamiliar	0.71 ± 0.41	0.76 ± 0.41	**0.0187**	**0.0001**
Song	Mate	0.66 ± 0.41	0.68 ± 0.42		
	Familiar	0.79 ± 0.41	0.70 ± 0.42	**0.0001**	0.3855
	Unfamiliar	0.78 ± 0.38	0.71 ± 0.40	**0.0004**	0.2033

Supplementary table 2

Latency of females' calls to males' playback. The mean ± the standard deviation of each playback series is reported together with the probabilities (P) that calls emitted during mate series have a longer latency than calls emitted during the other familiarity categories. The statistical significant differences are reported in bold.

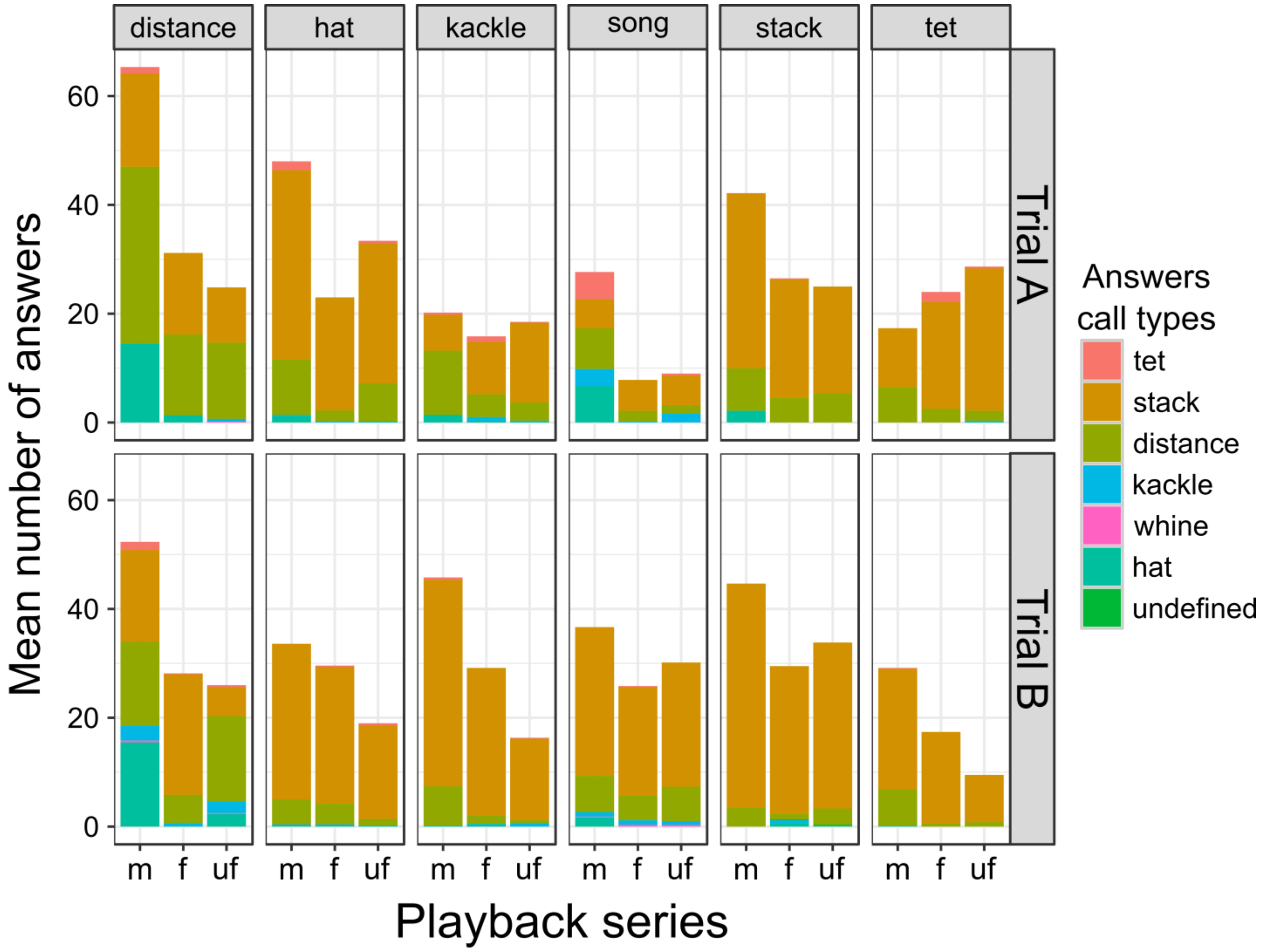

Supplementary fig. 2

Call types that females used to answer male playback. Shown are the mean number of answers for each playback series in trials A and B. Colours represent the type of answering call (used). Familiarity categories: **m**= mate of the focal bird; **f**= familiar individual; **uf**= unfamiliar individual.

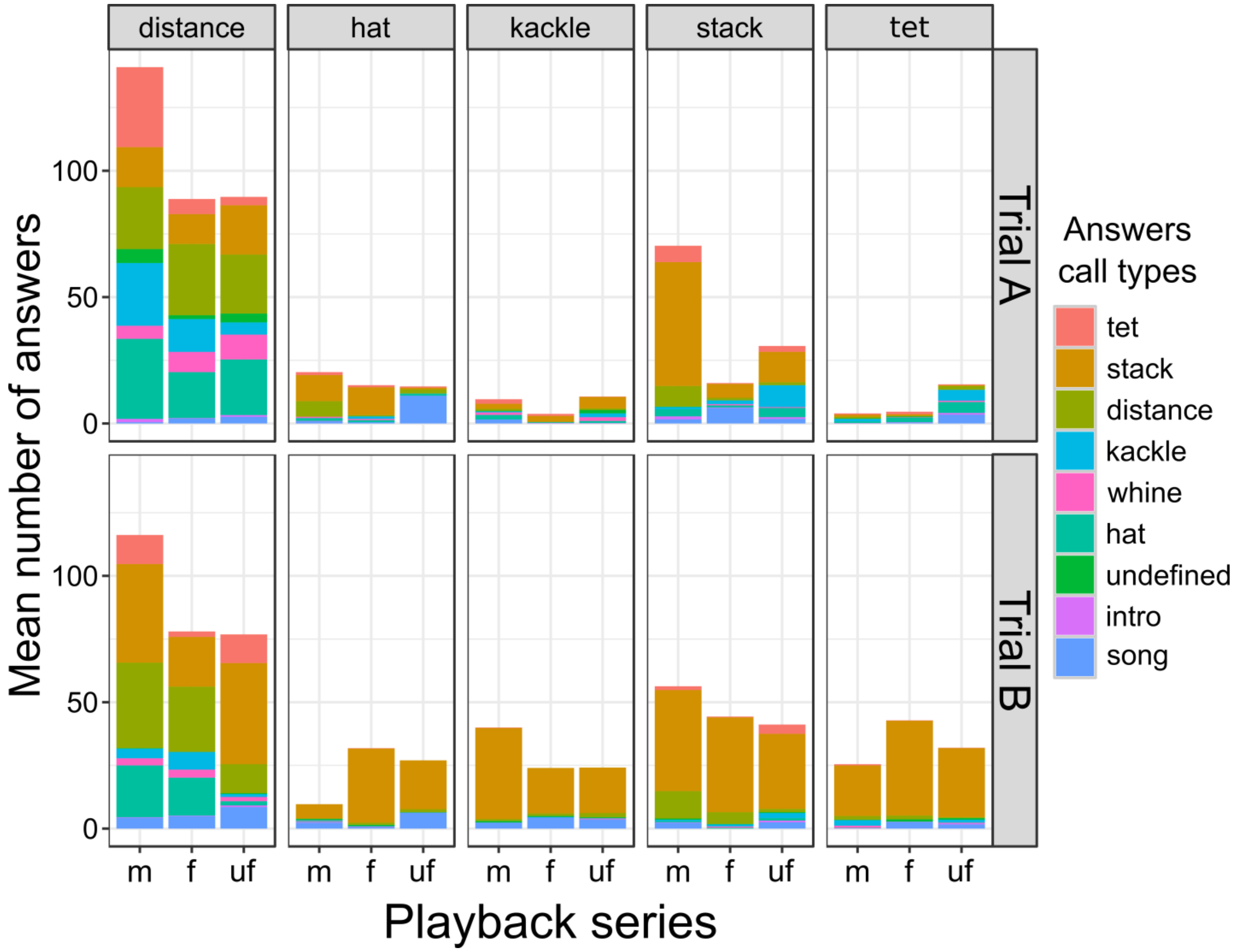

Supplementary fig. 3

Call types that males used to answer female playback. Shown are the mean number of answers for each playback series in trials A and B. Colours represent the type of answering vocalization (used) ("intro" = song introductory syllable). Familiarity categories: **m**= mate of the focal bird; **f**= familiar individual; **uf**= unfamiliar individual.

**Trial**	**Playback type**	**Answer type**	**Proportion of the specific call type used as answer (mean ± SD)**			**Test (N; K-W chi-squared; P-value)**
			Mate	Familiar	Unfamiliar	
A	Distance	Stack	0.31 ± 0.30	0.38 ± 0.30	0.42 ± 0.13	13; 0.7; 0.699
		Distance	0.54 ± 0.19	0.52 ± 0.32	0.56 ± 0.13	13; 0.03; 0.987
B	Distance	Stack	0.47 ± 0.28	0.75 ± 0.14	0.47 ± 0.38	13; 1.7; 0.418
		Distance	0.30 ± 0.22	0.16 ± 0.04	0.34 ± 0.37	13; 0.3; 0.875
A	Hat	Stack	0.67 ± 0.22	0.90 ± 0.08	0.69 ± 0.24	13; 5.4; 0.067
		Distance	0.28 ± 0.18	0.07 ± 0.09	0.25 ± 0.13	13; 5.4; 0.068
B	Hat	Stack	0.89 ± 0.09	0.89 ± 0.10	0.93 ± 0.06	9; 1.1; 0.576
A	Kackle	Stack	0.39 ± 0.37	0.52 ± 0.36	0.80 ± 0.18	15; 3.2; 0.200
		Distance	0.51 ± 0.38	0.26 ± 0.23	0.19 ± 0.18	15; 2; 0.365
B	Kackle	Stack	0.87 ± 0.10	0.94 ± 0.04	0.84 ± 0.23	15; 0.9; 0.630
		Distance	0.12 ± 0.10	0.05 ± 0.04	0.06 ± 0.07	15; 1.8; 0.397
A	Song	Distance	0.34 ± 0.32	0.37 ± 0.18	0.16 ± 0.11	8; 1.4; 0.488
B	Song	Stack	0.62 ± 0.21	0.87 ± 0.13	0.84 ± 0.16	10; 3; 0.223
A	Stack	Stack	0.86 ± 0.16	0.82 ± 0.12	0.77 ± 0.23	12; 0.7; 0.692
		Distance	0.11 ± 0.14	0.18 ± 0.12	0.23 ± 0.23	12; 1.4; 0.488
B	Stack	Stack	0.93 ± 0.08	0.92 ± 0.03	0.95 ± 0.07	15; 0.8; 0.673
		Distance	0.07 ± 0.08	0.04 ± 0.03	0.05 ± 0.07	15; 0.7; 0.692
A	Tet	Stack	0.68 ± 0.32	0.72 ± 0.41	0.82 ± 0.40	16; 2.9; 0.237
		Distance	0.32 ± 0.32	0.08 ± 0.09	0.05 ± 0.08	16; 5.5; 0.065
B	Tet	Stack	0.80 ± 0.20	0.98 ± 0.04	0.81 ± 0.32	12; 2.4; 0.307

Supplementary table 3

Females' answers to males' playback of the three familiarities. Represented are the over threshold (at least 5 calls per bird per series, at least 8 non-null values per series) tested proportions of each answer type over all the answers for each series. A numerical example illustrates best our approach: to test whether birds used differential proportions of stacks out of all the answers to respond to play-backed stacks depending on the familiarity level we did the follow: If for example a bird used 30 calls to answer the stack mate calls and 15 of these answers were stacks, its proportion of answers with stack to mate's stack was 50%; if the same bird then used 20 calls to answer the familiar stacks and 15 of these answers were stacks, its proportion of answers with stacks to familiar stacks was 75%; finally, if the same bird used 20 calls to answer the unfamiliar stacks and 18 of these answers were stacks, its proportion of answers with stacks to unfamiliar stacks was 90%. We did the same for every tested bird and then compared the proportion of the three familiarities with a non-parametric test (Kruskal-Wallis rank sum test).

Trial	Playback type	Answer type	Proportion of the specific call type used as answer (mean ± SD)			Test (N; K-W chi-squared; P-value)
			Mate	Familiar	Unfamiliar	
A	Distance	Tet	0.22 ± 0.24	0.08 ± 0.10	0.03 ± 0.05	18; 5.3; 0.070
		Stack	0.11 ± 0.16	0.27 ± 0.33	0.25 ± 0.21	18; 0.9; 0.641
		Distance	0.20 ± 0.19	0.28 ± 0.35	0.27 ± 0.26	18; 0.04; 0.982
		Whine	0.03 ± 0.04	0.15 ± 0.27	0.17 ± 0.37	18; 0.04; 0.980
		Hat	0.23 ± 0.27	0.09 ± 0.17	0.13 ± 0.26	18; 2; 0.372
B	Distance	Tet	0.06 ± 0.10	0.03 ± 0.04	0.14 ± 0.34	18; 0.2; 0.904
		Stack	0.31 ± 0.26	0.49 ± 0.44	0.51 ± 0.39	18; 1.1; 0.567
		Distance	0.25 ± 0.21	0.21 ± 0.33	0.14 ± 0.13	18; 1; 0.612
		Whine	0.04 ± 0.08	0.03 ± 0.05	0.03 ± 0.03	18; 0.4; 0.829
		Hat	0.19 ± 0.37	0.15 ± 0.24	0.03 ± 0.05	18; 0.5; 0.781
A	Hat	Stack	0.55 ± 0.25	0.64 ± 0.44	0.07 ± 0.08	9; 2.2; 0.326
B	Hat	Stack	0.62 ± 0.31	0.92 ± 0.13	0.75 ± 0.20	11; 2.7; 0.255
A	Kackle	Stack	0.36 ± 0.34	0.67 ± 0.18	0.44 ± 0.30	9; 1.5; 0.472
B	Kackle	Stack	0.93 ± 0.06	0.82 ± 0.23	0.78 ± 0.26	15; 0.6; 0.754
		Distance	0.02 ± 0.02	0.03 ± 0.06	0.05 ± 0.05	15; 0.4; 0.820
A	Stack	Stack	0.64 ± 0.14	0.57 ± 0.41	0.57 ± 0.47	13; 0.1; 0.952
		Distance	0.13 ± 0.16	0.04 ± 0.07	0.04 ± 0.04	13; 1; 0.601
B	Stack	Stack	0.72 ± 0.25	0.85 ± 0.08	0.69 ± 0.31	17; 0.4; 0.816
		Distance	0.14 ± 0.23	0.10 ± 0.09	0.05 ± 0.05	17; 0.8; 0.666
B	Tet	Stack	0.83 ± 0.25	0.87 ± 0.07	0.81 ± 0.29	13; 1; 0.621

Supplementary table 4

Males' answers to females' playback of the three familiarities. Represented are the over threshold (at least 5 calls per bird per series, at least 8 non-null values per series) tested proportions of each answer type over all the answers for each series. For further explanation and a numerical example see caption of the table S3

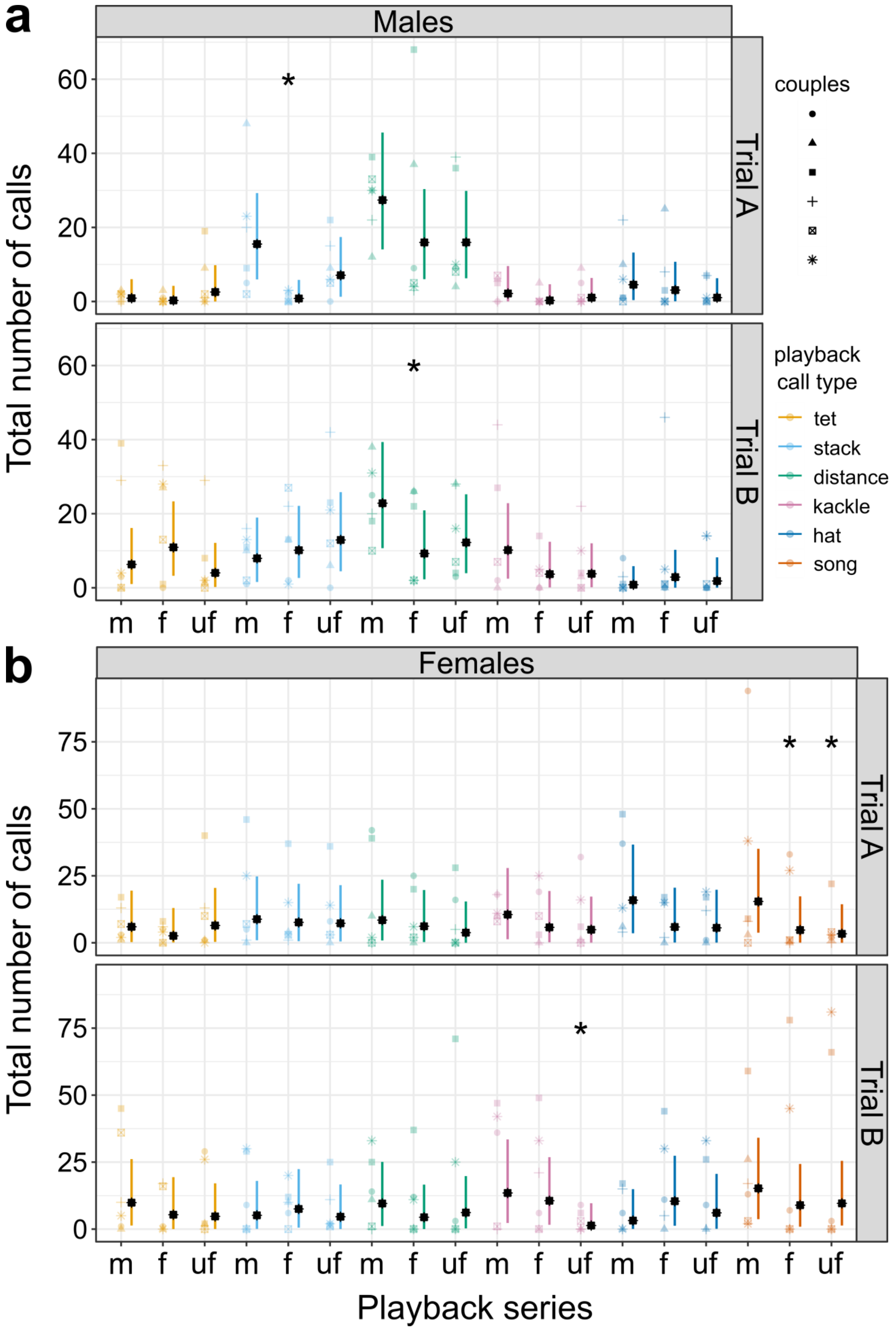
a
Males
Trial A
Trial B
Total number of calls
couples
playback call type
tet
stack
distance
kackle
hat
song
m
f
uf
b
Females
Playback series

Supplementary fig. 4

Number of answering calls that focal individuals emitted during the broadcasting of the first 30 playback stimuli of the different playback series. For males (**A**) and females (**B**) and both trials raw data (symbols indicate responses of individual birds) and the computed 95% credible intervals (error bars) as well as the fitted value (black symbols) are shown. Colours represent the type of playback call broadcast; symbols indicate responses of individual birds. Familiarity categories: **m**= mate of the focal bird; **f**= familiar individual; **uf**= unfamiliar individual; stars mark significant differences from the category "mate".

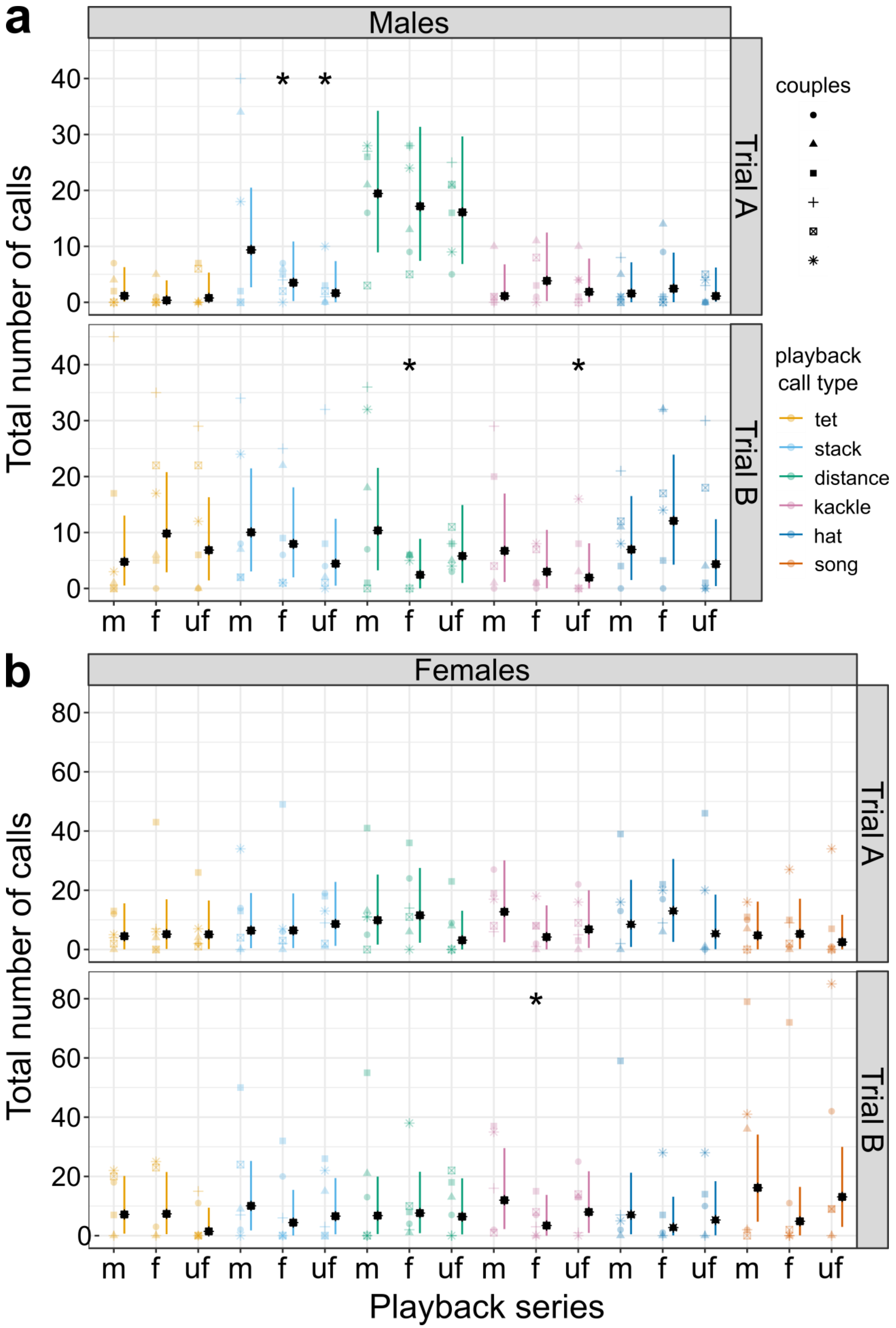

a
Males
Trial A
Trial B
Total number of calls
couples
playback call type
tet
stack
distance
kackle
hat
song
m f uf m f uf m f uf m f uf m f uf
b
Females
Trial A
Trial B
Total number of calls
m f uf m f uf m f uf m f uf m f uf m f uf
Playback series

Supplementary fig. 5

Number of answering calls that focal individuals emitted during the broadcasting of the last 30 playback stimuli of the different playback series. For males (**A**) and females (**B**) and both trials raw data (symbols indicate responses of individual birds) and the computed 95% credible intervals (error bars) as well as the fitted value (black symbols) are shown. Colours represent the type of playback call broadcast; symbols indicate responses of individual birds. Familiarity categories: **m**= mate of the focal bird; **f**= familiar individual; **uf**= unfamiliar individual; stars mark significant differences from the category "mate".

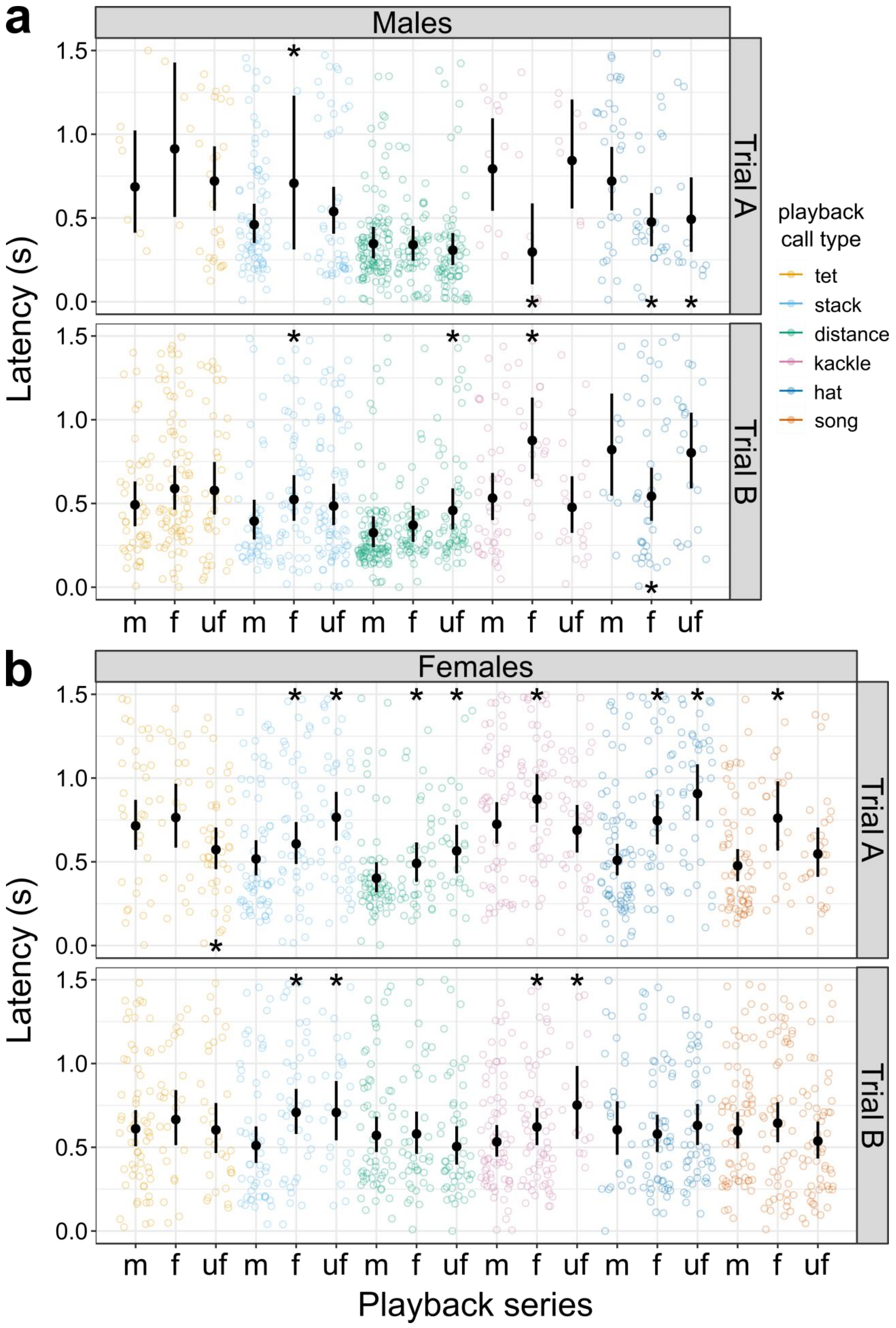
a
Males
Trial A
Trial B
Latency (s)
playback call type
tet
stack
distance
kackle
hat
song
m f uf m f uf m f uf m f uf m f uf
b
Females
Trial A
Trial B
Latency (s)
m f uf m f uf m f uf m f uf m f uf m f uf
Playback series

Supplementary fig. 6

Latency to the first answering call, measured for the first 30 (playback) stimuli of the different playback series (analysed time interval: 0 - 1.5 s after the onset of the playback stimulus). Colours represent the type of playback call broadcast; dots indicate individual calls (raw data). Familiarity categories: **m**= mate of the focal bird; **f**= familiar individual; **uf**= unfamiliar individual. For males (**A**) and females (**B**) and both trials the computed 95% credible intervals (error bars) as well as the fitted value (black symbols) are shown. Stars mark significant differences from the category "mate"; stars at the bottom indicate significance in the direction opposite to the expectation (i.e. slower answers to the mate than to non-mates).

Call type	Familiarity	Latency		P mate slower		Qualitatively consistent with full dataset?	
		A	B	A	B	A	B
Tet	Mate	0.80 ± 0.43	0.49 ± 0.33				
	Familiar	1.04 ± 0.42	0.65 ± 0.41	0.0836	0.0862	YES	YES
	Unfamiliar	0.67 ± 0.44	0.63 ± 0.44	0.4116	0.1147	YES	YES
Stack	Mate	0.52 ± 0.34	0.41 ± 0.32				
	Familiar	0.62 ± 0.55	0.61 ± 0.39	**0.0001**	**0.0247**	NO	YES
	Unfamiliar	0.56 ± 0.46	0.53 ± 0.39	0.0954	0.0799	YES	NO
Distance	Mate	0.37 ± 0.27	0.33 ± 0.26				
	Familiar	0.32 ± 0.20	0.36 ± 0.22	0.4982	0.4974	YES	YES
	Unfamiliar	0.32 ± 0.31	0.49 ± 0.36	0.8049	**0.0042**	YES	NO
Kackle	Mate	0.84 ± 0.40	0.57 ± 0.39				
	Familiar	0.53 ± 0.59	0.87 ± 0.32	**1**	**0**	NO	NO
	Unfamiliar	0.92 ± 0.30	0.51 ± 0.37	0.3716	0.7859	YES	YES
Hat	Mate	0.83 ± 0.45	0.80 ± 0.39				
	Familiar	0.55 ± 0.36	0.61 ± 0.46	**0.9968**	**0.9757**	NO	NO
	Unfamiliar	0.47 ± 0.43	0.84 ± 0.41	**0.995**	0.5419	NO	YES

Supplementary table 5

Latency of males' calls to females' playback considering only the first 30 calls of each series. The mean ± the standard deviation of each playback series is reported together with the probability (P) value that calls emitted during mate series have a longer latency than calls emitted during the other familiarity categories. The statistical significant differences are reported in bold. The two rightmost columns indicated whether the result is different from the one obtained considering the entire playback series.

Call type	Familiarity	Latency		P mate slower		Qualitatively consistent with full dataset?	
		A	B	A	B	A	B
Tet	Mate	0.81 ± 0.43	0.66 ± 0.40				
	Familiar	0.83 ± 0.40	0.74 ± 0.38	0.2611	0.1715	YES	NO
	Unfamiliar	0.62 ± 0.33	0.68 ± 0.41	**0.9758**	0.5432	NO	NO
Stack	Mate	0.57 ± 0.37	0.59 ± 0.41				
	Familiar	0.70 ± 0.42	0.77 ± 0.40	**0.0462**	**0.0002**	YES	YES
	Unfamiliar	0.76 ± 0.39	0.75 ± 0.38	**0**	**0.0001**	YES	YES
Distance	Mate	0.44 ± 0.31	0.62 ± 0.38				
	Familiar	0.52 ± 0.30	0.58 ± 0.37	**0.0271**	0.8984	YES	NO
	Unfamiliar	0.60 ± 0.33	0.55 ± 0.36	**0.0001**	0.431	YES	YES
Kackle	Mate	0.77 ± 0.42	0.58 ± 0.33				
	Familiar	0.91 ± 0.39	0.61 ± 0.36	**0.0107**	**0.0394**	NO	YES
	Unfamiliar	0.72 ± 0.37	0.79 ± 0.33	0.7197	**0**	YES	YES
Hat	Mate	0.56 ± 0.38	0.67 ± 0.40				
	Familiar	0.82 ± 0.42	0.62 ± 0.38	**0**	0.6271	YES	YES
	Unfamiliar	0.95 ± 0.39	0.65 ± 0.35	**0**	0.3757	YES	NO
Song	Mate	0.52 ± 0.33	0.65 ± 0.35				
	Familiar	0.74 ± 0.34	0.71 ± 0.44	**0**	0.2027	YES	YES
	Unfamiliar	0.59 ± 0.34	0.55 ± 0.36	0.0775	0.8752	NO	YES

Supplementary table 6

Latency of females' calls to males' playback considering only the first 30 calls of each series. The mean ± the standard deviation of each playback series is reported together with the probability (P) value that calls emitted during mate series have a longer latency than calls emitted during the other familiarity categories. The statistical significant differences are reported in bold. The two rightmost columns indicated whether the result is different from the one obtained considering the entire playback series.

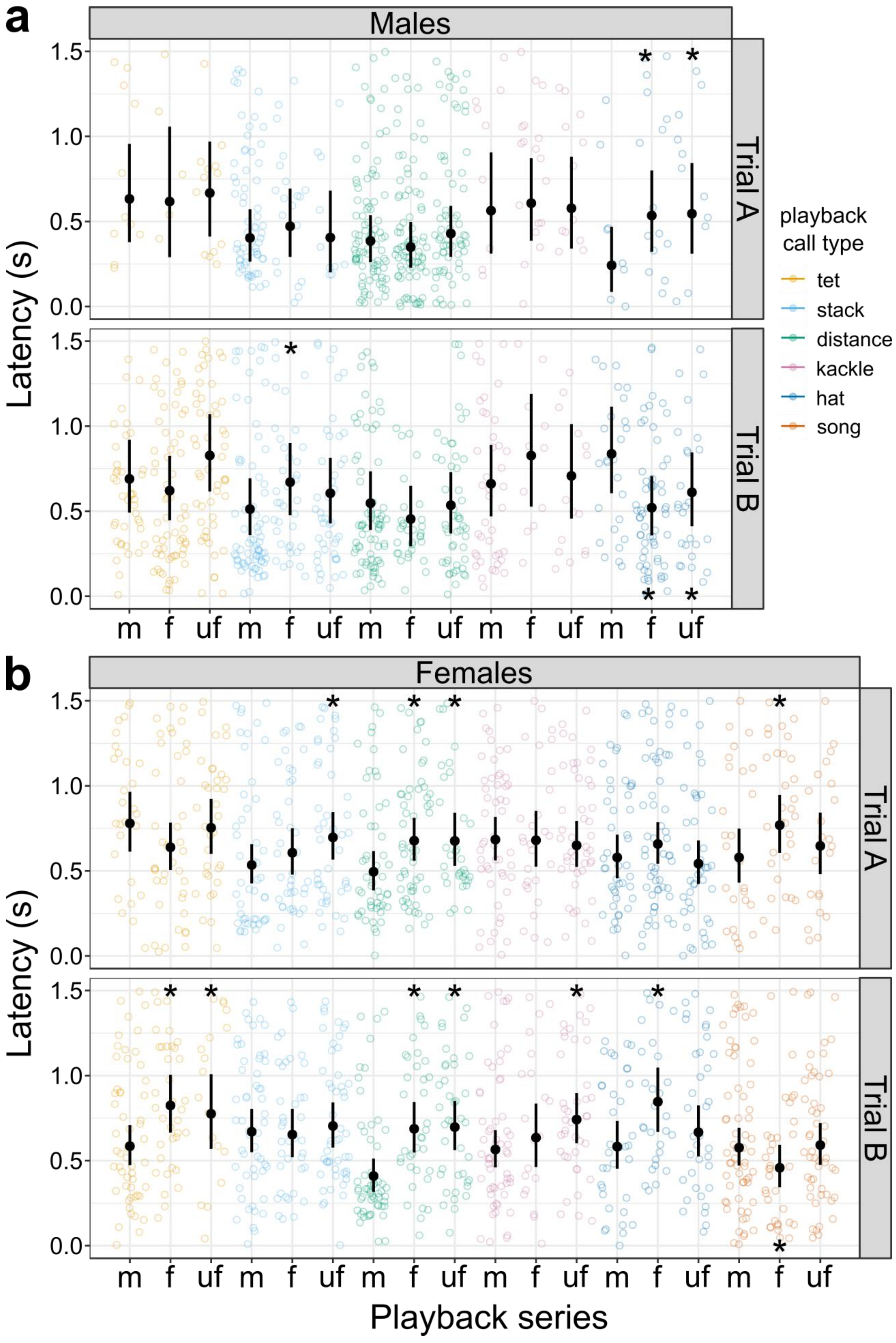

a
Males
Trial A
Trial B
Latency (s)
0.0
0.5
1.0
1.5
m
f
uf
playback call type
tet
stack
distance
kackle
hat
song
b
Females
Playback series

Supplementary fig. 7

Latency to the first answering call, measured for the last 30 (playback) stimuli of the different playback series (analysed time interval: 0 - 1.5 s after the onset of the playback stimulus). Colours represent the type of playback call broadcast; dots indicate individual calls (raw data). Familiarity categories: **m**= mate of the focal bird; **f**= familiar individual; **uf**= unfamiliar individual. For males (**A**) and females (**B**) and both trials the computed 95% credible intervals (error bars) as well as the fitted value (black symbols) are shown. Stars mark significant differences from the category "mate", stars at the bottom indicate significance in the direction opposite to the expectation (i.e. slower answers to the mate than to non-mates).

Call type	Familiarity	Latency		P mate slower		Qualitatively consistent with full dataset?	
		A	B	A	B	A	B
Tet	Mate	0.77 ± 0.47	0.61 ± 0.32				
	Familiar	0.87 ± 0.45	0.60 ± 0.42	0.5469	0.7434	YES	YES
	Unfamiliar	0.66 ± 0.34	0.80 ± 0.42	0.4131	0.1148	YES	YES
Stack	Mate	0.56± 0.37	0.50 ± 0.42				
	Familiar	0.58 ± 0.36	0.63 ± 0.37	0.2021	**0.0414**	YES	YES
	Unfamiliar	0.50 ± 0.37	0.59 ± 0.42	0.4894	0.1476	YES	NO
Distance	Mate	0.47 ± 0.36	0.52 ± 0.38				
	Familiar	0.44 ± 0.34	0.42 ± 0.24	0.5022	0.506	YES	YES
	Unfamiliar	0.56 ± 0.41	0.54 ± 0.38	0.2751	0.5683	YES	YES
Kackle	Mate	0.85 ± 0.49	0.63 ± 0.44				
	Familiar	0.80 ± 0.40	0.81 ± 0.42	0.3835	0.0706	YES	YES
	Unfamiliar	0.75 ± 0.38	0.62 ± 0.40	0.4619	0.3243	YES	YES
Hat	Mate	0.43 ± 0.41	0.77 ± 0.39				
	Familiar	0.75 ± 0.51	0.52 ± 0.37	**0.0076**	**0.9977**	NO	NO
	Unfamiliar	0.75 ± 0.45	0.60 ± 0.40	**0.0063**	**0.9721**	NO	NO

Supplementary table 7

Latency of males' calls to females' playback considering only the last 30 calls of each series. The mean ± the standard deviation of each playback series is reported together with the probability (P) value that calls emitted during mate series have a longer latency than calls emitted during the other familiarity categories. The statistical significant differences are reported in bold. The two rightmost columns indicated whether the result is different from the one obtained considering the entire playback series.

Call type	Familiarity	Latency		P mate slower		Qualitatively consistent with full dataset?	
		A	B	A	B	A	B
Tet	Mate	0.85 ± 0.42	0.67 ± 0.40				
	Familiar	0.70 ± 0.46	0.83 ± 0.38	0.9476	**0.0001**	YES	YES
	Unfamiliar	0.80 ± 0.38	0.68 ± 0.37	0.6155	**0.0021**	YES	YES
Stack	Mate	0.60 ± 0.45	0.72 ± 0.39				
	Familiar	0.64 ± 0.40	0.69 ± 0.36	0.1143	0.6098	NO	NO
	Unfamiliar	0.74 ± 0.42	0.73 ± 0.37	**0.0054**	0.2941	YES	NO
Distance	Mate	0.58 ± 0.39	0.45 ± 0.34				
	Familiar	0.71 ± 0.37	0.73 ± 0.38	**0.0021**	**0**	YES	YES
	Unfamiliar	0.69 ± 0.36	0.74 ± 0.38	**0.0023**	**0**	YES	NO
Kackle	Mate	0.73 ± 0.40	0.62 ± 0.39				
	Familiar	0.72 ± 0.41	0.73 ± 0.45	0.5208	0.1153	YES	NO
	Unfamiliar	0.73 ± 0.35	0.79 ± 0.37	0.6942	**0.0021**	YES	YES
Hat	Mate	0.65 ± 0.37	0.61 ± 0.38				
	Familiar	0.72 ± 0.42	0.83 ± 0.36	0.1217	**0.0004**	NO	NO
	Unfamiliar	0.57 ± 0.41	0.75 ± 0.43	0.7051	0.1229	NO	NO
Song	Mate	0.66 ± 0.46	0.66 ± 0.43				
	Familiar	0.84 ± 0.39	0.49 ± 0.38	**0.0138**	**0.9869**	YES	NO
	Unfamiliar	0.68 ± 0.41	0.63 ± 0.37	0.2061	0.3962	NO	YES

Supplementary table 8

Latency of females' calls to males' playback considering only the last 30 calls of each series. The mean ± the standard deviation of each playback series is reported together with the probability (P) value that calls emitted during mate series have a longer latency than calls emitted during the other familiarity categories. The statistical significant differences are reported in bold. The two rightmost columns indicated whether the result is different from the one obtained considering the entire playback series.

call_comb.	ans_m	awd_m	ans_f	awd_f	day	ans_m_p	awd_m_p	ans_f_p	awd_f_p	cp
stack_stack	148	125	124	152	d1	38.54	32.55	21.12	25.89	1
stack_stack	175	174	184	177	d3	33.27	33.08	21.47	20.65	1
stack_stack	208	243	243	203	d1	15.77	18.42	19.35	16.16	2
stack_stack	296	267	262	293	d3	15.23	13.74	16.21	18.13	2
stack_stack	1196	1346	1173	1045	d1	32.88	36.99	20.94	18.65	5
stack_stack	1041	1302	1274	1009	d3	29.77	37.23	23.20	18.38	5
stack_stack	70	84	86	75	d1	7.88	9.46	7.21	6.29	8
stack_stack	193	224	227	188	d3	4.06	4.71	16.62	13.76	8
stack_stack	79	87	91	81	d1	15.52	17.09	2.03	1.80	9
stack_stack	169	196	198	180	d3	22.84	26.49	4.06	3.69	9
stack_stack	64	127	120	60	d1	10.261	20.35	5.60	2.80	11
stack_stack	333	462	450	323	d3	12.97	17.99	6.60	4.75	11

Supplementary table 9

Stack-stack call interactions in all six focal pairs during the baseline (days 1 and 3). Absolute and relative numbers of answered stack calls and stack calls used as answers by males and females (ans_m, ans_f: number of stack calls that were used by male or female, respectively, to answer a partner's stack call; awd_m, awd_f: number of male or female stack calls, respectively, that were answered by a stack call of the partner; ans_m_p, ans_f_p: percentaged number of stack calls that were used by male or female, respectively, to answer a partner's stack call; awd_m_p, awd_f_p: percentage number of male or female stack calls, respectively, that were answered by a stack call of the partner; cp: couple ID).

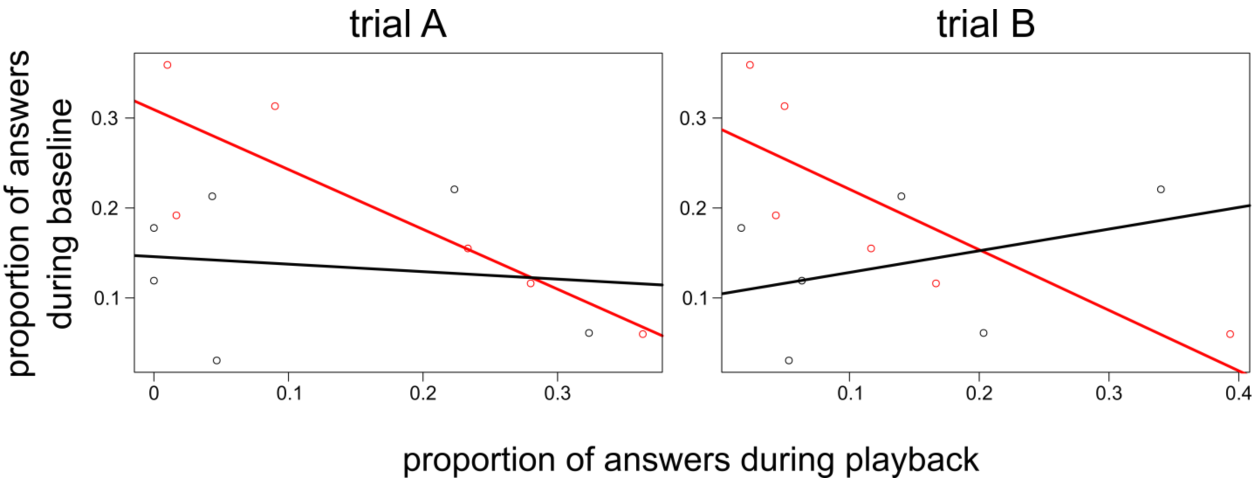

Tria A, MALES, Baseline~playback experiment			
	**Estimate**	**Standard Error**	**P-value**
Intercept	3.09	0.44	
playback experiment	-6.66	2.07	**0.0324**
**Tria B, MALES, Baseline~playback experiment**			
Intercept	2.88	0.45	
playback experiment	-6.73	2.46	0.0523
**Tria A, FEMALES, Baseline~playback experiment**			
Intercept	1.45	0.47	
playback experiment	-0.83	2.93	0.7905
**Tria B, FEMALES, Baseline~playback experiment**			
Intercept	1.04	0.54	
playback experiment	2.41	3.09	0.4784

Supplementary fig. 8

Relationship between the proportion of stack-stack answers given during baseline (mean of both days) and those during trials A and B of the playback experiment. Dots represent individual birds, lines the regression line of the linear model between the 2 variables, whose results are reported in the table together with the correspondent P-value. The males are depicted in red and the females in black.

a

Sex	Call	Mean	SD
m	Distance	1.9%	4.5%
m	Hat	0	0
m	Kackle	20%	9.3%
m	Song	5.6%	6.1%
m	Tet	7.4%	9.1%
m	Stack	7.4%	13.5%
f	Distance	20.4%	14.8%
f	Hat	1.9%	4.5%
f	Kackle	13.9%	5.6%
f	Tet	3.2%	5.4%
f	Stack	7.4%	9.1%

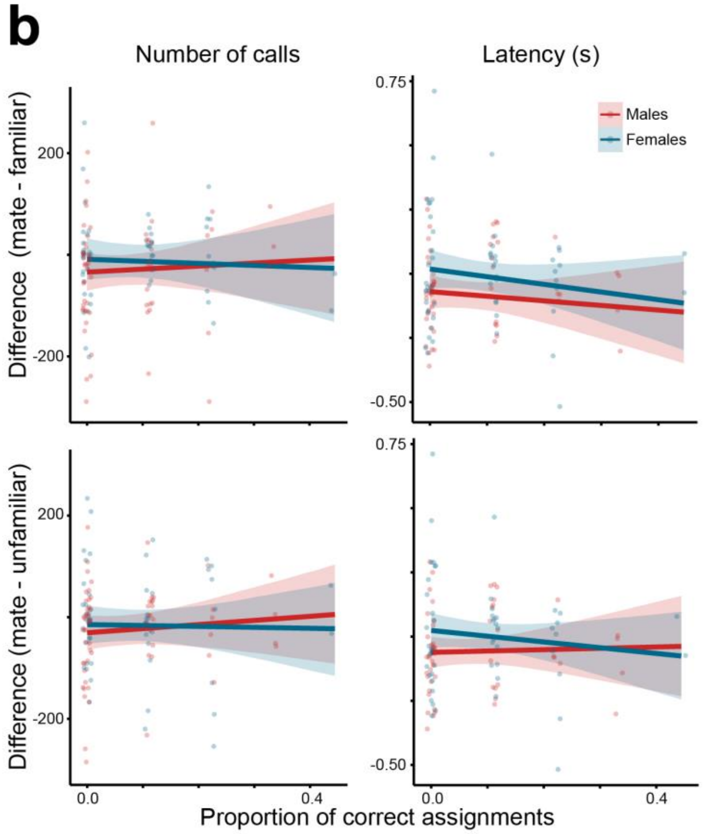

Supplementary fig. 9

Relation between the variability in the call type (e.g., stack call) to the variation in conspecific response. (a) Values of incorrect assignment of the linear discriminant analysis. For each call type the percentage of incorrectly assigned calls are reported (mean value and the standard deviation, n=6 individuals per sex). Higher values correspond to more similar calls (i.e. call types more difficult to assign to the correct individual). (b) Results of linear mixed model (shaded areas) with row data (dots) correlating the within call type variability as a predictor of the conspecific response (number of calls left panel and latency in the right panels). Top panels contain the differences between mate and familiar and the bottom panels between mate and unfamiliar. Male calls are depicted in red and females in blues.
